# Ab initio determination of crystal stability of di-p-tolyl disulfide

**DOI:** 10.1038/s41598-021-86519-1

**Published:** 2021-03-29

**Authors:** Xuan Hao, Jinfeng Liu, Imran Ali, Hongyuan Luo, Yanqiang Han, Wenxin Hu, Jinyun Liu, Xiao He, Jinjin Li

**Affiliations:** 1 School of Chemistry and Molecular Engineering, Shanghai Engineering Research Center of Molecular Therapeutics and New Drug Development, East China Normal University, Shanghai, 200062 China; 2 Key Laboratory for Thin Film and Microfabrication of Ministry of Education, Department of Micro/Nano-Electronics, Shanghai Jiao Tong University, Shanghai, 200240 China; 3 State Key Laboratory of Natural Medicines, Department of Basic Medicine and Clinical Pharmacy, China Pharmaceutical University, Nanjing, 210009 China; 4 School of Computer Science and Software Engineering, The Computer Center, East China Normal University, Shanghai, 200062 China; 5 Anhui Provincial Engineering Laboratory for New-Energy Vehicle Battery Energy-Storage Materials, Anhui Laboratory of Molecule-Based Materials, College of Chemistry and Materials Science, Anhui Normal University, Wuhu, 241002 Anhui China; 6 NYU-ECNU Center for Computational Chemistry at NYU Shanghai, Shanghai, 200062 China

**Keywords:** Chemical engineering, Density functional theory, Structure prediction

## Abstract

With the rapid growth of energy demand and the depletion of existing energy resources, the new materials with superior performances, low costs and environmental friendliness for energy production and storage are explored. Di-p-tolyl disulfide (*p*-Tol_2_S_2_) is a typical lubricating material, which has been applied in the field of energy storage. The conformational properties and phase transformations of *p*-Tol_2_S_2_ have been studied by pioneers, but their polymorphs and the polymorphism induced crystal structure changes require further analysis. In this study, we perform the crystal structural screening, prediction and optimization of *p*-Tol_2_S_2_ crystal with quantum mechanical calculations, i.e., density functional theory (DFT) and second-order Møller–Plesset perturbation (MP2) methods. A series of crystal structures with different molecular arrangements are generated based on the crystal structure screening. As compared to long-established lattice energy calculation, we take an advantage of using more accurate technique, which is Gibbs free energy calculation. It considers the effects of entropy and temperature to predict the crystal structures and energy landscape. By comparing the Gibbs free energies between predicted and experimental structures, we found that phase α is the most stable structure for *p*-Tol_2_S_2_ crystal at ambient temperature and standard atmospheric pressure. Furthermore, we provide an efficient method to discriminate different polymorphs that are otherwise difficult to be identified based on the Raman/IR spectra. The proposed work enable us to evaluate the quality of various crystal polymorphs rapidly.

## Introduction

**Figure 1:**
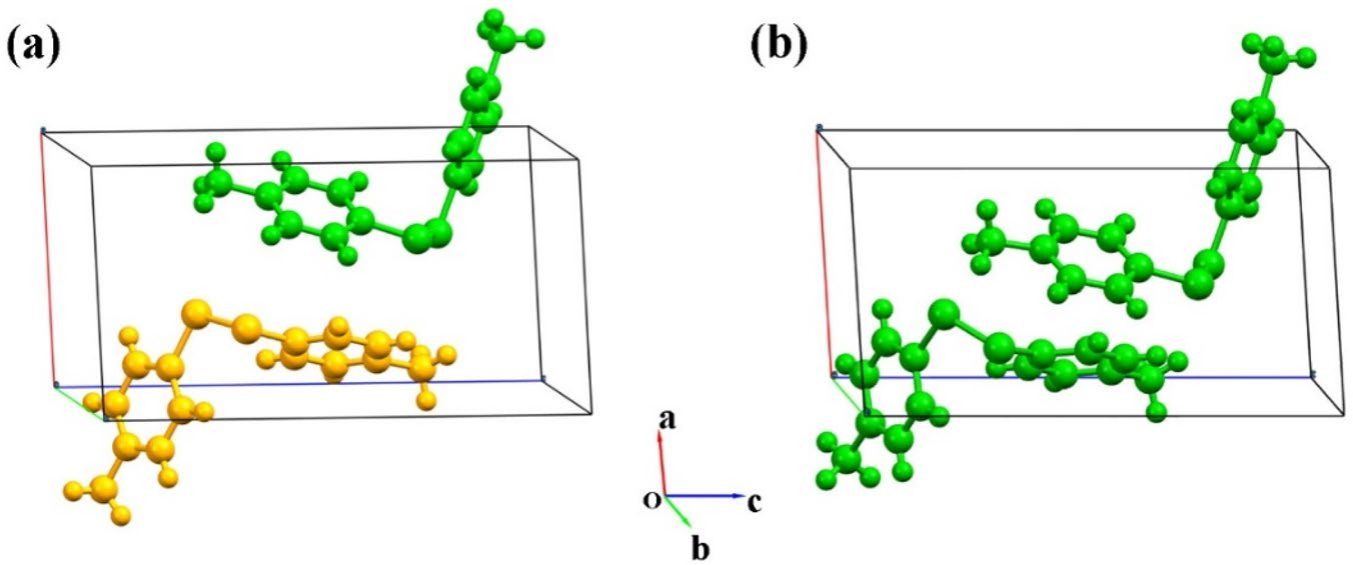
Crystal structures of *p*-Tol_2_S_2_. Phase α (**a**) has a space group of P2_1_, while phase β (**b**) has a space group of P_1_. The molecules are colored by the symmetry operation, where the unit cells of phase α and phase β contain one and two independent molecules, respectively.

Organic disulfide has been one of the major interesting research materials due to its superior lubricating property^1^ and the extensive applications in storing energy^2^. Di-p-tolyl disulfide (*p*-Tol_2_S_2,_ with the formula of C_14_H_14_S_2_), a typical representative molecule of a large group of diphenyl disulfides, has been explored in many aspects^3–5^. A number of analyses have examined the interplay of conformational transformation and the thermodynamic stability of the *p*-Tol_2_S_2_, demonstrating that the change in pressure^6–9^ or recrystallization^10–12^ would result in structural phase transition. Previous studies have primarily concentrated on the pressure effect on the conformational qualities of *p*-Tol_2_S_2_ and its molecular aggregation in crystalline state^13–17^, but the polymorphs and polymorphism induced difference either in the crystal structure or the physical and chemical properties have not been studied. The present paper focuses on the polymorphs of *p*-Tol_2_S_2_, studying the stability of different forms and providing an effective spectral method to distinguish them. There are several different polymorphs of *p*-Tol_2_S_2_ with different conformations and molecular aggregations. The two main structures are phase α and phase β. Phase α is an independent molecule (Z′ = 1), which has an asymmetric conformation, with the monoclinic unit cell of space group P2_1_. At higher pressures (> 1.6 GPa), phase α transform into a steric hindrance and aggregated phase β with reduced lattice parameters and volume. Structure β is a triclinic phase with two independent molecules (Z′ = 2) per unit cell and has a space group of P_1_ (see Fig. 1). The structures of *p-*Tol_2_S_2_ were determined by X-ray diffraction and its cell parameters can be found in the Cambridge Structural Database (CSD).

Polymorphisms, differing in their physical properties, have different solubility and dissolution rates. Therefore, discrimination of polymorphisms is important for all molecular crystal products and industrial production. Crystal structure prediction (CSP) is an emerging tool to help crystallographers determine various thermodynamically favorable crystal packings which are independent of the kinetics of crystallization. However they depend on a wide range of controllable or less easily controllable crystallization condition^18–20^. The advancement and development of CSP have been widely performed on different polymorphisms^21–23^. In this paper, to obtain more reliable structures, we use different CSP tools of MOLPAK^24,25^ (MOLecular PAcKing) and USPEX^26–28^ to perform crystal structure screening, prediction and optimization of *p*-Tol_2_S_2_. The MOLPAK and USPEX totally produce 9000+ possible hypothetical crystal structures which lie in the most common space groups with different molecular arrangements. The 38 candidates (20 from MOLPAK and 18 from USPEX) with the lowest lattice energies are selected to perform further optimization, where the crystal structure optimization is calculated at the DFT level using the ωB97XD/6-31G* method, and the single-point electronic energy is calculated at the MP2 level on the optimized crystal structure. The MP2 calculation is used to obtain more accurate and reliable prediction results. Besides, to treat the macromolecular system, such as the *p*-Tol_2_S_2_ (has 30 atoms) crystal in the present paper, the embedded-fragment method^29–31^ is used to obtain the total energy by taking an appropriate combination of the energies of monomers and dimers, which are embedded in the electrostatic field of the rest in the crystalline environment. Via the Gibbs free energy calculation, we determine that phase α is the most stable structure from those predicted and experimental structures.

## Methods

### Crystal structure predictions

The credibility of computing methods for crystal structure prediction has been improved over the past years. Here we use the MOLPAK and USPEX tools to determine the crystal forms from the molecular structure. MOLPAK can create unit cells that have the smallest volumes per molecule by allowing surrounding molecules to collapse along the three axes of a Cartesian coordinate system until a predetermined repulsion threshold with the central molecule is reached^32,33^. While the USPEX program, adapting the evolutionary algorithm that can find the stable structures for systems with various rigid molecular shapes^26,34^. The initial structures are generally generated by selected space groups with the initial known structures under atmospheric pressure. Structure relaxation is implemented from low to high precision using the VASP program. All the structures are examined and their initial sorting was performed based on lattice energy or enthalpy after full relaxation. However selected potential structures were resorted according to root-mean-square deviations (RMSDs), given in Tables S1 and S2.

### Structure optimization and energy calculation

After generation of the initial crystal structures using MOLPAK and USPEX, for further calculation, 20 structures from MOLPAK and 18 structures from USPEX were selected based on lower lattice energies and enthalpies, respectively. To minimize the enthalpy, we adopt quasi-Newton algorithm^35^ for the crystal structure optimization. Because of the large molecular size and the quantity of molecules in the supercell, the embedded-fragment method^30,31,36^ with ωB97XD/6-31G* was used for the calculation of enthalpy and Gibbs free energy of the *p*-Tol_2_S_2_ crystal. The Hessian matrix approximation was updated using the BFGS procedure^37^ and the convergence criterion for the maximum gradient was set to 0.001 Hartree/Bohr. The embedded fragment method, as the name implies, is a process that breaks a large system into small fragments. The embedded fragment method divides the total energy per unit cell of the crystal into an appropriate combination of the energies of monomers and dimers, and therefore can treat the macromolecules effectively. The individual molecule is designated as a segment, and the interaction energies between two segments, in close contact with each other, are calculated by quantum mechanics (QM). The interaction energy between the two distal segments is calculated according to Coulomb interactions. The DFT calculations, accounting for the environmental influences, are embedded in an electrostatic field represented by the point charges of the remaining system.

The internal energy of a unit cell for the molecular crystal system is calculated by:

1 $${\text{E}}_{e} = \mathop \sum \limits_{i} E_{i\left( 0 \right)} + \mathop \sum \limits_{{\begin{array}{*{20}c} {i,j,i < j} \\ {R_{ij} \le \lambda } \\ \end{array} }} \left( {E_{i\left( 0 \right)j\left( 0 \right)} - E_{i\left( 0 \right)} - E_{j\left( 0 \right)} } \right) + \frac{1}{2}\mathop \sum \limits_{N = - S}^{S} \left( {1 - \delta_{n0} } \right)\mathop \sum \limits_{{\begin{array}{*{20}c} {i,j} \\ {R_{ij} \le \lambda } \\ \end{array} }} \left( {E_{i\left( 0 \right)j\left( n \right)} - E_{i\left( 0 \right)} - E_{j\left( n \right)} } \right) + E_{{{\text{LR}}}} ,$$

where the three-integer index of unit cell is represented by a variable ‘*n’*. The quantum mechanical (QM) energy of the *i*th molecules in the *n*th unit cell is represented by ${ }E_{i\left( n \right)}$. The QM energy of dimers is represented by $E_{i\left( 0 \right)j\left( n \right)}$, where *i*th and *j*th represent number of molecules with respect to 0th central unit cell and *n*th unit cells^29,38–41^. The crystal system is represented by a $3 \times 3 \times 3$ supercell. The 1st part of Eq. (1) calculates all the single molecular energies in the *0*th unit cell, which is in the center. The 2nd part represents the two-body QM interaction that has a shorter distance than $\lambda$ (where $\lambda$ is a given cutoff distance which is set to 4 Å in this work). The 3rd part of Eq. (1) provides the interactions between one molecule in the central unit cell and the other in *n*th unit cell whose distance is shorter than $\lambda$. The QM is used to calculate short range interactions (the first three parts in Eq. (1)). It is calculated in electrostatic field of the rest, where ωB97XD/6-31G* level is used to fit electrostatic potential variations. The background charges are represented by the $11 \times 11 \times 11$ supercell. The Coulomb’s charge-charge interaction is employed to approximately treat long-range interactions between two molecules of dimers whose distance is larger than $\lambda$. The long-range electrostatic interactions are represented by $E_{{{\text{LR}}}}$ in a $41 \times 41 \times 41$ supercell.

The enthalpy $H_{e}$ for each unit cell can be calculated by considering the effect of external pressure as follow:

2 $$H_{e} = E_{e} + PV,$$

where the external pressure is represented by *P* and the unit cell volume is represented by *V*.

3 $$U_{v} = \frac{1}{K}\mathop \sum \limits_{n} \mathop \sum \limits_{{\mathbf{k}}} \omega_{{n{\mathbf{k}}}} \left( {\frac{1}{2} + \frac{1}{{e^{{\beta \omega_{{n{\mathbf{k}}}} }} - 1}}} \right),$$

4 $$S_{v} = \frac{1}{\beta TK}\mathop \sum \limits_{n} \mathop \sum \limits_{{\mathbf{k}}} \left\{ {\frac{{\beta \omega_{{n{\mathbf{k}}}} }}{{e^{{\beta \omega_{{n{\mathbf{k}}}} }} - 1}} - {\text{ln}}\left( {1 - e^{{ - \beta \omega_{{n{\mathbf{k}}}} }} } \right)} \right\},$$

5 $$G_{e} = H_{e} + U_{v} - TS_{v} .$$

The harmonic approximation is used to calculate zero-point vibrational energy $U_{v}$ and the entropy ${ }S_{v}$, which are shown in Eqs. (3) and (4), respectively, where the frequency of phonon with lattice vector **k** is represented by $\omega_{{n{\mathbf{k}}}}$. $\beta = 1/k_{0} T$ and $k_{0}$ is the Boltzmann constant. The capital *K* is the product of all **k**, which are evenly spaced grid points in the reciprocal unit cell. The **k**-grid of 21 $\times$ 21 $\times$ 21 has been used in this study, where *K* = 9261. The calculation of Gibbs free energy with effects of temperature and pressure in a unit cell is given by Eq. (5).

As MP2/6-31G* is more accurate than DFT/ωB97XD/6-31G* but its computational time and cost are very high. Therefore, to be more efficient and effective, we optimized crystal structure as well as calculated enthalpy (*H*_*e*_) over single point energy, zero-point vibrational energy ($U_{v}$), and entropy ($S_{v}$) at DFT/ωB97XD/6-31G* level. Moreover, to obtain better accuracy, MP2/6-31G* level was used to recalculate enthalpy (*H*_*e*_) only, over single point energy based on crystal structure calculated by DFT/ ωB97XD/6-31G*.

### Raman spectra simulation

For a periodic molecular system, the dynamical force constant matrix is calculated as follow:

6 $${\text{D}}\left( {{\text{r}}_{p} ,{\text{r}}_{q} ,k} \right) = \frac{1}{{\sqrt {m_{p} m_{q} } }}\mathop \sum \limits_{n = - R}^{R} H\left( {r_{{{\text{p}},0}} ,{\text{r}}_{{{\text{q}},{\text{n}}}} } \right)e^{ - ikR\left( n \right)} ,$$

where the masses of atoms p and q are represented by *m*_*p*_ and *m*_*q*_, respectively.

The **k** is a Brillouin zone point which is known. The 2nd order derivative of the total energy in a unit cell which correspond to atom p and q in the *0*th and the *n*th cell, is represented by H(r_p,0_, r_q,n_) in Eq. (6) at geometry equilibrium. The number of nearby unit cells for QM treatment was truncated at R = 1, and the number of k-points was set to 21 in each of the three dimensions. The vibrational frequencies and the corresponding normal modes of the periodic molecular system can be obtained by solving the dynamical matrix ${\text{D}}\left( {{\text{r}}_{p} ,{\text{r}}_{q} ,k} \right)$. The zone-center (**k** = 0) vibrations presenting nonzero intensities results in the activation of IR- and Raman spectra. The force-constant matrix D(**0**) was used to calculate the vibrational frequency. Therefore, we can obtain the IR intensity (*I*_*n*0_) and Raman intensity (*R*_*n*0_) for the fundamental transition of the mode in the *n*th phonon branch with wave vector **k** = 0, demonstrated by the following derivatives:

7 $$I_{n0} \propto \mathop \sum \limits_{i}^{l,m,n} \left( {\frac{{\partial \mu_{i} }}{{\partial Q_{n0} }}} \right)^{2} ,$$

8 $$R_{n0} \propto \frac{3}{2}\left( {\mathop \sum \limits_{i}^{l,m,n} \frac{{\partial \alpha_{ii} }}{{\partial Q_{n0} }}} \right)^{2} + \frac{21}{2}\mathop \sum \limits_{i}^{l,m,n} \mathop \sum \limits_{j}^{l,m,n} \left( {\frac{{\partial \alpha_{ij} }}{{\partial Q_{n0} }}} \right)^{2} .$$

The corresponding normal mode is represented by *Q*_*n0*_. The dipole moment derivative ${ }\partial \mu_{i} /\partial Q_{n0}$ and the polarizability derivative ${ }\partial \alpha_{ij} /\partial Q_{n0}$ in the central unit cell can be derived with the embedded-fragment quantum mechanical method.

## Results and discussion

The experimental data of the *p*-Tol_2_S_2_ (CH_3_-C_6_H_4_-S)_2_ in the Cambridge Structural Database^42,43^ is used to predict the crystal structure. Phase α has a space group of P2_1_, with a monoclinic unit cell and a volume of 628.741 Å^3^. The cell lattice parameters are *a* = 7.5927 Å, *b* = 5.71318 Å, *c* = 14.722 Å, *α* = 90°, *β* = 94.7615°, *γ* = 90°. However, phase β has a space group of P1 with a unit cell volume of 545.963 Å^3^. The lattice parameters of unit cell are *a* = 7.3057 Å, *b* = 5.5093 Å, *c* = 14.038 Å, α = 95.14°, β = 97.23°, γ = 85.36°. Figure 1 represents the molecular structures of both (α and β) phases.

### Crystal structure predictions

The crystal structure prediction was performed by the MOLPAK and USPEX programs. From MOLPAK, we obtained 31 standard clusters of crystal structures with common space groups of (P2_1_2_1_2_1_, P$\overline{1}$, P2_1_/c, Cc, C2/c, P2_1,_ Pna2_1_ and Pbca). Each cluster contains 300+ crystal structures. Therefore, MOLPAK generated total crystal structures more than 9000. From these 9000+ crystal structures, only 20 crystal structures were selected according to lower lattice energies, and these selected structures were further optimized. All the selected 20 structures from MOLPAK have same lattice energy of − 16.91 kcal/mol, given in Table S1. We obtained same lattice energy for 20 selected structures because of two main reasons: first, these structures are similar to each other with minor difference in their lattice parameters, and second, the MOLPAK software does not calculate lattice energy very accurately. Therefore, to obtain accurate energy landscape, we optimized selected structures and calculated their Gibbs free energy at the QM level.

On the other hand, USPEX generated 20 generations with each generation generated about 20 crystal structures. Therefore, the total crystal structures generated by USPEX were about 400. From USPEX predictions, only 18 crystal structures were selected based on enthalpy sorting. After selection of potential candidates we sorted them again, according to RMSDs, given in Table S2. The lattice parameters of phase α, phase β and the 38 predicted new crystal structures by MOLPAK and USPEX are given in Tables S1 and S2 as Supporting Information.

### The Gibbs free energy

**Figure 2:**
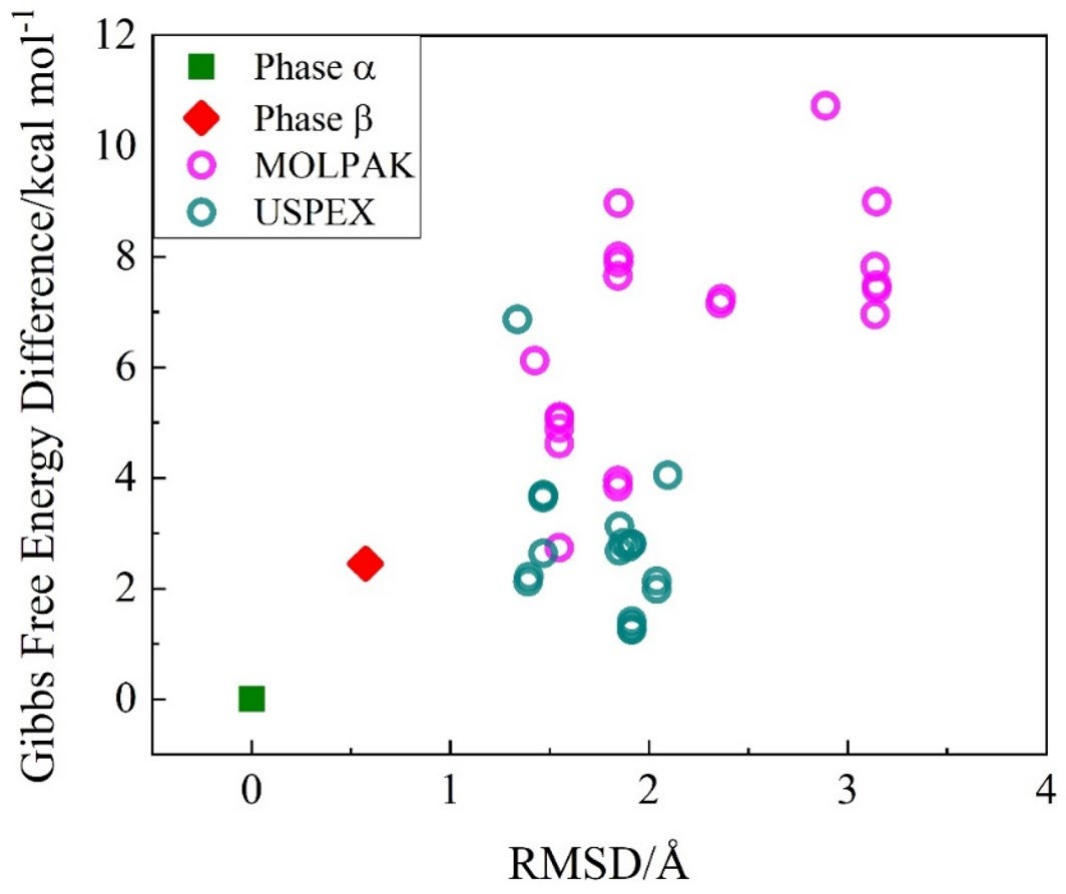
The Gibbs free energy differences per unit cell between the predicted crystal structures and experimental phase *α* as a function of the unit cell RMSD (with respect to phase *α*) under 298 K and standard atmospheric pressure, where the purple and green circles signify the 20 MOLPAK and 18 USPEX predicted structures, respectively. The green square and red diamond represent Gibbs free energies of the phases α and β, respectively, which were obtained from experiment.

The stability of 40 crystal structures are compared in Fig. 2, including phase α, phase β and 38 predicted new structures by MOLPAK and USPEX. Figure 2 shows the difference of Gibbs free energy per unit cell among 38 predicted structures, phase α and phase β of the *p*-Tol_2_S_2_ as a function of unit cell RMSD. In Fig. 2, the purple and green circles represent the 38 predicted structures by MOLPAK and USPEX, whereas the green square and red diamond denote the experimental Gibbs free energies of phases α and β, respectively. The enthalpy over single point energy of 40 crystal structures was calculated at the MP2/6-31G* level based on the DFT/ωB97XD/6-31G* optimized crystal structures.

The values of RMSD vary from 1 to 3.5 Å, which suggest that the crystal structure prediction method is reliable. Moreover, the lowest value of RMSD is smaller than 2 Å demonstrating the accuracy of prediction. The correlation between RMSD and Gibbs free energy (as shown in Fig. 2) reveals that the free energy of phase α, locating at the bottom of the lattice energy landscape, is the most stable structure among all predicted and experimental structures. Furthermore, the selected 20 structures from MOLPAK were possessing same lattice energies, given in Table S1. However, in Fig. 2 Gibbs free energy varies from about 1.5 to 8 kcal/mol, which is evidence of accuracy of Gibbs free energy calculation.

To verify the rationality of predicted structures, we selected eight candidates from 38 predicted structures by USPEX and MOLPAK programs to visualize overlay only. Figures S1 and S2 in the Supporting Information present the similarity of crystal structures and the molecular conformations via the calculations of the structural RMSDs, phase α and the predicted structures based on the RDKIT tool^44^. We selected a subset of 8 structures from 38 structures based on difference in RMSDs. The green molecules represent the structures of *p*-Tol_2_S_2_ phase α determined by X-ray diffraction and the red molecules denote the predicted structures based on USPEX and MOLPAK programs. The molecular overlay shows the reliability of the predicted structures.

### Vibrational spectra

**Figure 3:**
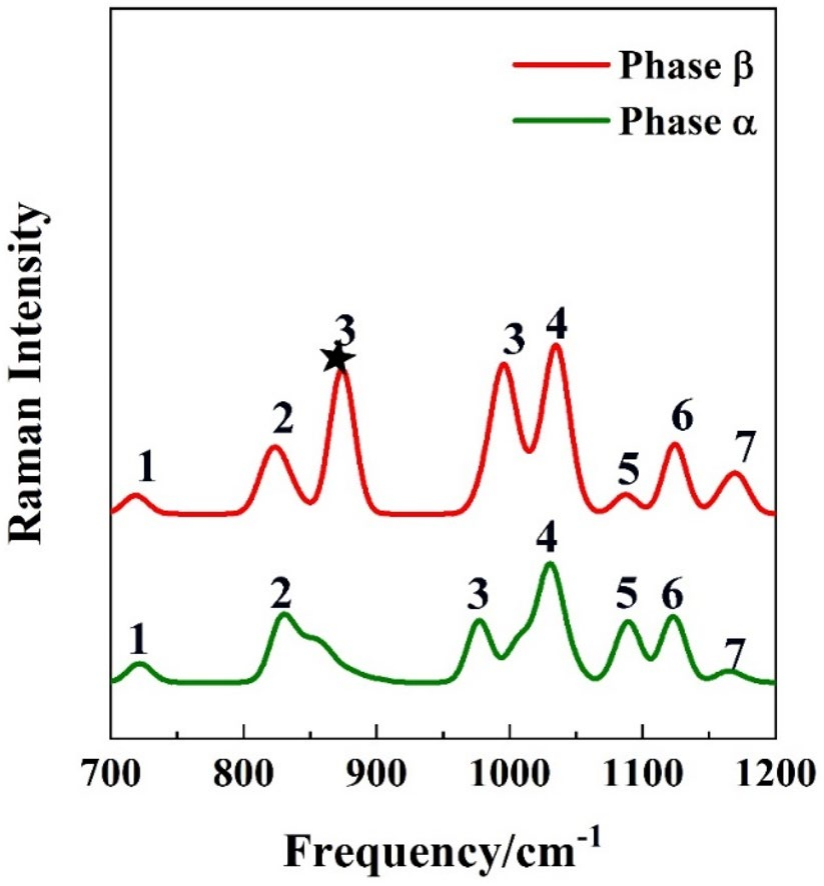
Calculated Raman spectra of *p*-Tol_2_S_2_ phase α (green) and phase β (red) under standard atmospheric pressure.

**Figure 4:**
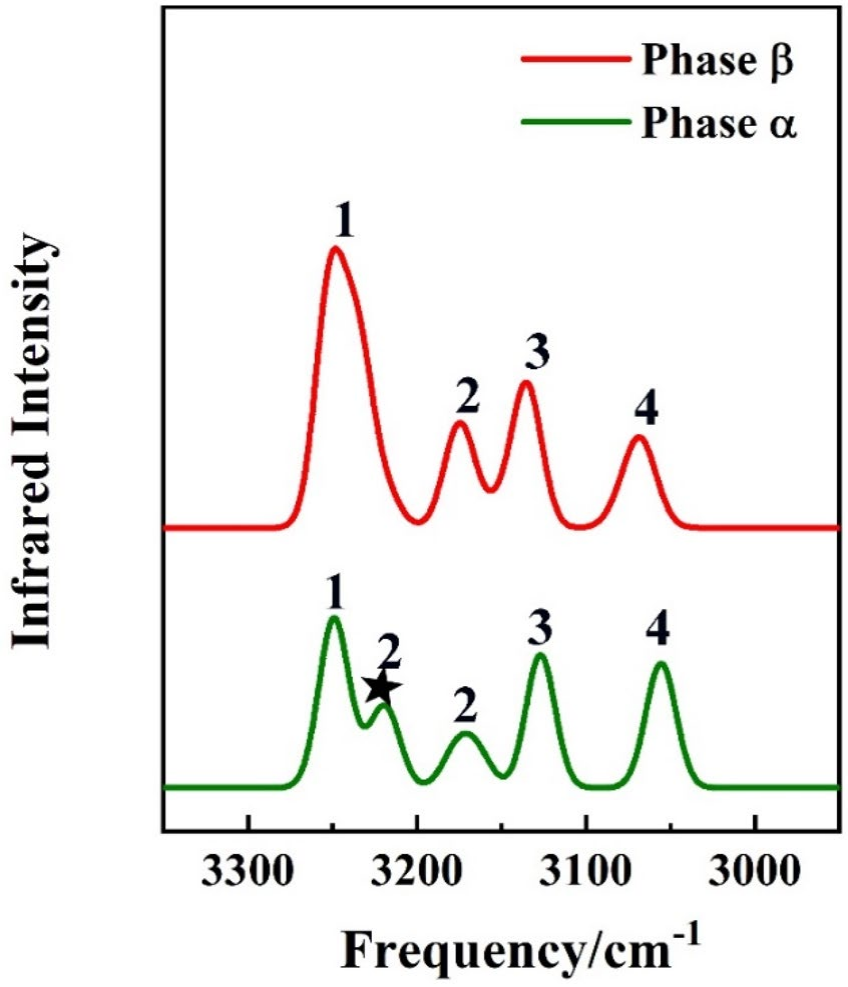
Calculated IR spectra of *p*-Tol_2_S_2_ phase α (green) and phase β (red) under standard atmospheric pressure.

The crystal structure of a molecule can be identified by vibrational spectroscopy^45,46^. The calculated full range of Raman spectra are provided in Figs. S3 and S4 of the Supporting Information. For the purpose of highlighting the differences of phase α and phase β, we select parts of the calculated Raman and IR spectra of *p*-Tol_2_S_2_ crystals. The Raman and IR spectra are shown in Figs. 3 and 4. In Fig. 3, the Raman spectra are calculated from 700 to 1200 cm^−1^, where phase α (green curve) and phase β (red curve) are predicted to display seven and eight discernible intense Raman bands, respectively. The band 3*, locating at ~ 874 cm^–1^, in the red curve is the characteristic peak for phase β. Similarly, there are five and four IR bands for phase α (green curve) and phase β (red curve), respectively. The band 2*, locating at ~ 3220 cm^−1^, in the green curve is the characteristic peak for phase α. Therefore, the band 3* in the Raman spectra and the band 2* in the IR spectra, are the characteristic bands to distinguish phases α and β of *p*-Tol_2_S_2_. Figures 3 and 4 show that although the structures of phases α and β are very similar (as shown in Fig. 1), they can be identified by the characteristic bands in the vibrational spectra. The vibrational spectra of different pressure about phase α and phase β are demonstrated in Figs. S5–S8 in the Supporting Information. We can observe the vibrational spectra as a function of different pressures.

## Conclusions

It is highly desirable to control polymorph for all the manufacturing industries which manufacture and/or utilize crystalline products. This will enable us to enhance functionality of the products to next higher level. A high precision method to screen different polymorphs for designing of crystals and to find out the most stable structure that confers an optimal product would, therefore, be extremely valuable. In this paper, we carried out a theoretical approach to screen different polymorphs of *p*-Tol_2_S_2_, a lubricating and energy storage material, based on the DFT and MP2 methods with the embedded fragment approach. With the theoretical calculation, we pick out the most stable structure phase α from the predicted and experimental structures. Considering the small RMSDs, there will be hundreds of predicted structures between them and the conformation of existing phases α and β, the proposed method has successfully performed the predictions for sufficiently similar conformations along with the identification of the most stable structure. The predicted vibrational spectra provide an effective way to identify polymorphs with the characteristic peaks of IR and Raman spectra.

## Supplementary Information

Supplementary Information.
