# Supplementary material for: Ab initio determination of crystal stability of di-p-tolyl disulfide
Source: Sci Rep. 2021 Mar 29;11:7076. doi: 10.1038/s41598-021-86519-1 (PMC8007795; doi:10.1038/s41598-021-86519-1)
Supplement: Supplementary file 1 — Supplementary Information. [file 41598_2021_86519_MOESM1_ESM.docx]

**Supporting Information for**

***Ab initio* Determination of Crystal stability of**

**Di-p-tolyl Disulfide**

Xuan Hao^1,2†^, Jinfeng Liu^3†^, Imran Ali^2†^, Hongyuan Luo^2^, Yanqiang Han^2^, Wenxin Hu^4^, Jinyun Liu^5*^, Xiao He^1,6*^ and Jinjin Li^2*^

*^1^Shanghai Engineering Research Center of Molecular Therapeutics and New Drug Development, School of Chemistry and Molecular Engineering, East China Normal University, Shanghai, 200062, China*

*^2^Key Laboratory for Thin Film and Microfabrication of Ministry of Education, Department of Micro/Nano-electronics, Shanghai Jiao Tong University, Shanghai 200240, China.*

*^3^State Key Laboratory of Natural Medicines, Department of Basic Medicine and Clinical Pharmacy,* *China Pharmaceutical University, Nanjing, 210009, China*

*^4^The Computer Center, School of Computer Science and Software Engineering, East China Normal University, Shanghai 200062, China*

*^5^Key Laboratory of Functional Molecular Solids of the Ministry of Education, Anhui Laboratory of Molecule-Based Materials, College of Chemistry and Materials Science, Anhui Normal University, Wuhu, Anhui 241000, China*

*^6^NYU-ECNU Center for Computational Chemistry at NYU Shanghai, Shanghai, 200062, China*

^*^Correspondence to: Jinjin Li ([lijinjin@sjtu.edu.cn](mailto:lijinjin@sjtu.edu.cn)) (J.L.), Jinyun Liu ([jyliu@ahnu.edu.cn](mailto:jyliu@ahnu.edu.cn)) (J.L.) and Xiao He ([xiaohe@phy.ecnu.edu.cn](mailto:xiaohe@phy.ecnu.edu.cn)) (X.H.)

^†^These authors contributed equally to this work.

To compare the predicted structures of *p*-Tol_2_S_2_ by MOLPAK and USPEX with experiment, this Supporting Information provides the comparisons of lattice parameters and RMSD of the molecular overlay between calculation and experiment. Tables S1 and S2 are the lattice parameters and RMSD comparisons between predicted structures and experiment. We select eight predicted structures from the predicted 38 candidates to perform the structural overlap with the experimental phase α, which are shown in Figs. S1-S2. The lowest RMSDs using MOLPAK and USPEX are 1.424 and 1.337 Å, respectively, which are both lower than 2 Å, indicating the validity of the crystal structure prediction method. Figs.3-4 are the calculated Raman and IR spectra of phases α, β of *p*-Tol_2_S_2_, which can be used as a method of distinguishing two phases. Figs.S5-S8 are the calculated Raman and IR spectra of phases α, β of *p*-Tol_2_S_2_ with different pressures.

**Table S1**. Lattice parameters for the MOLPAK predicted and experimental structures of di-p-tolyl Disulfide (*p*-Tol_2_S_2_).

| Structure ID | Space group | *a* (Å) | *b* (Å) | *c* (Å) | *α* (deg) | *β* (deg) | | *γ* (deg) | Cell volume (Å**^3^)** | RMSD (Å) | Lattice Energy (kcal/mol) |
| --- | --- | --- | --- | --- | --- | --- | --- | --- | --- | --- | --- |
| Phase *α* | P2_1_ | 7.5927 | 5.71318 | 14.722 | 90 | | 94.7615 | 90 | 636.414 | 0 |  |
| Phase *β* | P1 | 7.3057 | 5.5093 | 14.038 | 95.14 | | 97.23 | 85.36 | 556.9 | 0.573 |  |
| 1 | P$\bar{1}$ | 6.9268 | 12.8028 | 8.9769 | 80.905 | | 67.471 | 74.344 | 706.72 | 1.424 | -16.91 |
| 2 | P$\bar{1}$ | 6.9268 | 12.8028 | 8.9766 | 80.905 | | 67.475 | 74.344 | 706.71 | 1.547 | -16.91 |
| 3 | P$\bar{1}$ | 6.9267 | 12.8028 | 8.9767 | 80.906 | | 67.475 | 74.344 | 706.71 | 1.547 | -16.91 |
| 4 | P$\bar{1}$ | 12.8027 | 6.9268 | 8.9762 | 112.519 | | 80.907 | 105.655 | 706.72 | 1.547 | -16.91 |
| 5 | P$\bar{1}$ | 12.8028 | 6.9268 | 8.9774 | 67.462 | | 80.902 | 74.343 | 706.71 | 1.549 | -16.91 |
| 6 | P$\bar{1}$ | 12.8028 | 6.9268 | 8.9765 | 67.477 | | 80.907 | 74.344 | 706.72 | 1.549 | -16.91 |
| 7 | P$\bar{1}$ | 6.9268 | 12.8028 | 8.9767 | 80.906 | | 112.526 | 105.656 | 706.72 | 1.843 | -16.91 |
| 8 | P$\bar{1}$ | 12.8027 | 6.9268 | 8.9956 | 67.186 | | 92.87 | 105.656 | 706.72 | 1.843 | -16.91 |
| 9 | P$\bar{1}$ | 6.9267 | 12.8074 | 8.9764 | 92.819 | | 67.478 | 105.729 | 706.71 | 1.843 | -16.91 |
| 10 | P$\bar{1}$ | 6.9268 | 12.8073 | 8.9767 | 87.181 | | 67.475 | 74.271 | 706.72 | 1.846 | -16.91 |
| 11 | P$\bar{1}$ | 6.9268 | 12.8074 | 8.9761 | 87.183 | | 67.483 | 74.27 | 706.71 | 1.846 | -16.91 |
| 12 | P$\bar{1}$ | 12.8073 | 6.9268 | 8.9767 | 67.474 | | 92.82 | 105.729 | 706.71 | 1.846 | -16.91 |
| 13 | P$\bar{1}$ | 6.9268 | 12.8028 | 8.9952 | 92.868 | | 112.809 | 74.344 | 706.72 | 2.357 | -16.91 |
| 14 | P$\bar{1}$ | 6.9268 | 12.8073 | 8.9766 | 87.181 | | 67.475 | 74.271 | 706.71 | 2.364 | -16.91 |
| 15 | P$\bar{1}$ | 5.8713 | 5.9388 | 23.5379 | 103.252 | | 97.187 | 106.947 | 747.78 | 2.888 | -16.91 |
| 16 | P$\bar{1}$ | 12.8074 | 6.9268 | 8.9767 | 112.526 | | 92.82 | 74.271 | 706.72 | 3.137 | -16.91 |
| 17 | P$\bar{1}$ | 12.8074 | 6.9268 | 8.9766 | 67.475 | | 92.82 | 105.729 | 706.7 | 3.137 | -16.91 |
| 18 | P$\bar{1}$ | 12.8073 | 6.9268 | 8.9768 | 112.528 | | 92.82 | 74.272 | 706.71 | 3.145 | -16.91 |
| 19 | P$\bar{1}$ | 12.8028 | 6.9268 | 8.9947 | 67.199 | | 92.867 | 105.657 | 706.71 | 3.145 | -16.91 |
| 20 | P$\bar{1}$ | 12.8027 | 6.9268 | 8.9958 | 67.184 | | 92.871 | 105.655 | 706.73 | 3.145 | -16.91 |

**Table S2**. Lattice parameters for the USPEX predicted and experimental structures of di-p-tolyl Disulfide (*p*-Tol_2_S_2_).

| Structure ID | Space group | *a* (Å) | *b* (Å) | *c* (Å) | *α* (deg) | *β* (deg) | *γ* (deg) | Cell volume (Å**^3^)** | RMSD (Å) | Enthalpy (eV) |
| --- | --- | --- | --- | --- | --- | --- | --- | --- | --- | --- |
| Phase *α* | P2_1_ | 7.5927 | 5.71318 | 14.722 | 90 | 94.7615 | 90 | 636.414 | 0 |  |
| Phase *β* | P1 | 7.3057 | 5.5093 | 14.038 | 95.14 | 97.23 | 85.36 | 556.9 | 0.573 |  |
| 1 | P1 | 7.5686 | 7.0531 | 12.0513 | 90.79 | 86.92 | 91.9 | 641.998 | 1.337 | -370.617 |
| 2 | P1 | 6.4421 | 7.2171 | 13.0431 | 90.08 | 90.24 | 88.3 | 606.147 | 1.391 | -370.488 |
| 3 | P1 | 6.4069 | 7.2728 | 13.0448 | 90.07 | 90.23 | 88.35 | 607.574 | 1.395 | -370.507 |
| 4 | P1 | 6.599 | 7.7167 | 12.8162 | 92.57 | 88.31 | 92.06 | 651.305 | 1.467 | -370.741 |
| 5 | P1 | 6.5984 | 7.7164 | 12.8161 | 92.57 | 88.31 | 92.07 | 651.207 | 1.467 | -370.742 |
| 6 | P1 | 6.5977 | 7.7161 | 12.8165 | 92.57 | 88.3 | 92.09 | 651.123 | 1.468 | -370.743 |
| 7 | P1 | 6.5973 | 7.7158 | 12.8161 | 92.56 | 88.3 | 92.1 | 651.039 | 1.468 | -370.744 |
| 8 | P1 | 6.2513 | 7.7723 | 12.8594 | 90.56 | 89.71 | 88.53 | 624.555 | 1.851 | -370.643 |
| 9 | P1 | 6.2516 | 7.7713 | 12.8613 | 90.55 | 89.71 | 88.53 | 624.596 | 1.851 | -370.644 |
| 10 | P2_1_ | 6.3283 | 7.0224 | 12.997 | 90 | 90 | 88.43 | 577.374 | 1.869 | -370.100 |
| 11 | P1 | 6.373 | 7.8199 | 12.92 | 91.2 | 89.18 | 90.35 | 643.667 | 1.897 | -370.788 |
| 12 | P1 | 6.5181 | 7.7308 | 12.7227 | 90.98 | 88.94 | 90.33 | 640.886 | 1.912 | -370.746 |
| 13 | P1 | 6.5177 | 7.7297 | 12.7235 | 90.97 | 88.95 | 90.34 | 640.803 | 1.913 | -370.747 |
| 14 | P1 | 6.5174 | 7.7289 | 12.7257 | 90.97 | 88.95 | 90.34 | 640.814 | 1.913 | -370.748 |
| 15 | P1 | 6.5173 | 7.7281 | 12.7269 | 90.96 | 88.95 | 90.35 | 640.805 | 1.913 | -370.749 |
| 16 | P1 | 6.3189 | 7.7760 | 12.8657 | 90.88 | 89.54 | 88.92 | 631.952 | 2.039 | -370.703 |
| 17 | P1 | 6.3187 | 7.7729 | 12.8657 | 90.88 | 89.54 | 88.92 | 631.758 | 2.039 | -370.704 |
| 18 | P1 | 6.5701 | 7.5565 | 12.3564 | 90.8 | 89.94 | 88.57 | 613.202 | 2.094 | -370.415 |
|  |  |  |  |  |  |  |  |  |  |  |


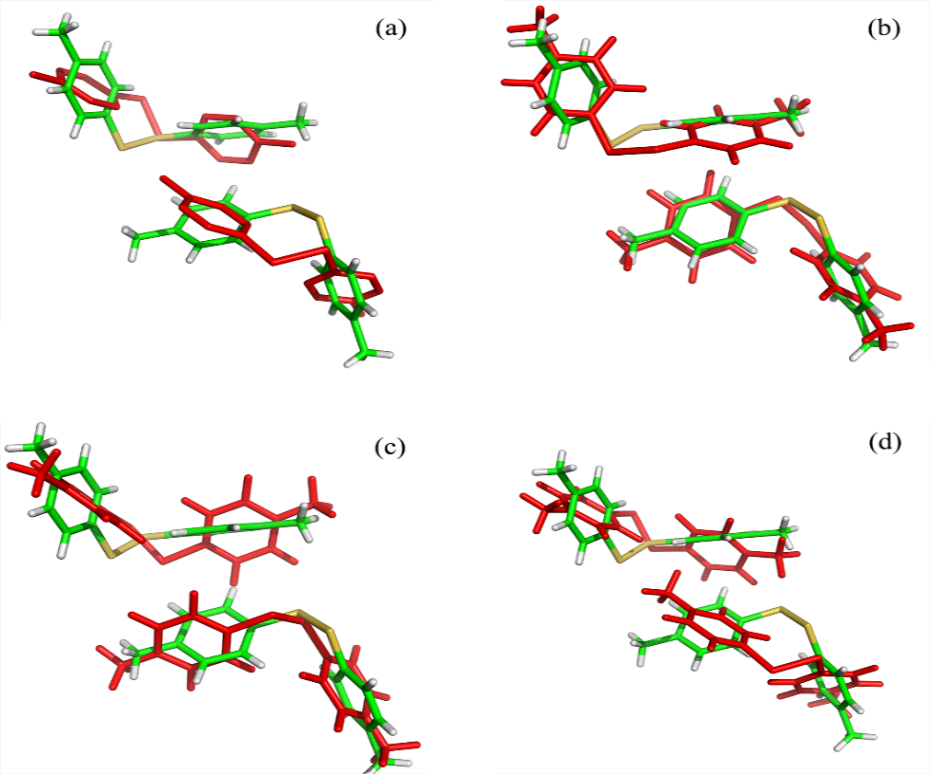


**Fig. S1** The root-mean-square deviation (RMSD) of molecule overlay using the RDKIT tool^1^. The green molecule is the structure of *p*-Tol_2_S_2_ phase *α* determined by X-ray diffraction and the red molecules are the predicted structures based on the MOLPAK program. The RMSD of (a), (b), (c) and (d) are 1.547, 1.424, 1.846 and 1.549 Å, respectively.


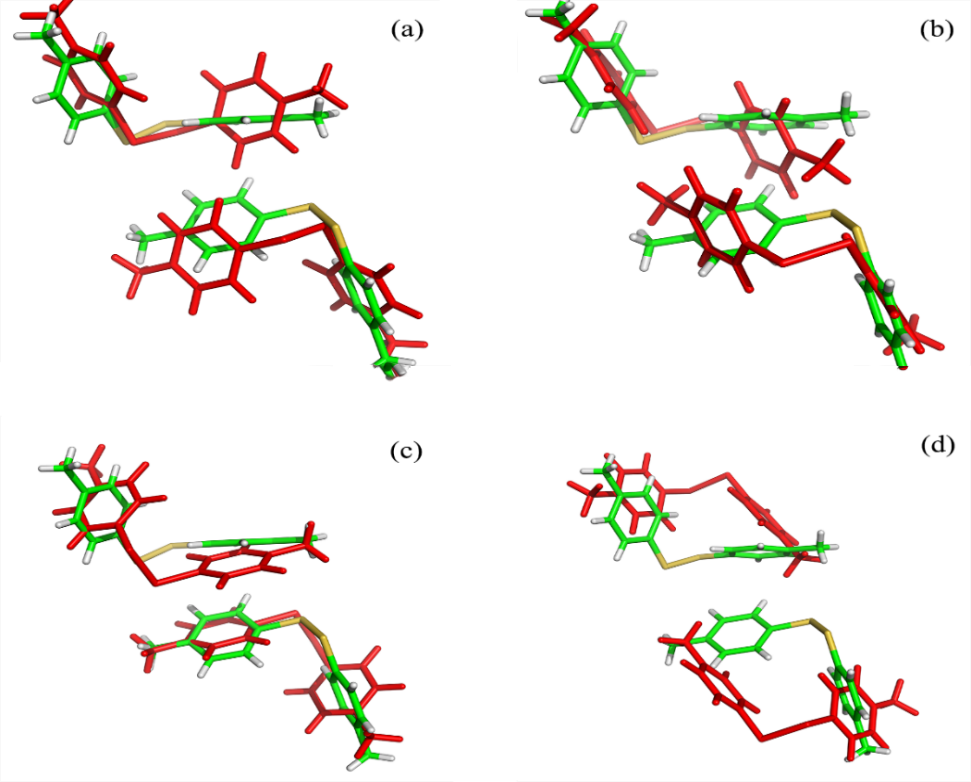


**Fig. S2** The root-mean-square deviation (RMSD) of molecule overlay using the RDKIT tool^1^. The green molecule is the structure of *p*-Tol_2_S_2_ phase *α* determined by X-ray diffraction and the red molecules are the predicted structures based on the USPEX program. The RMSD of (a), (b), (c) and (d) are 1.897, 1.337, 1.913 and 2.094 Å, respectively.

**Calculated Raman Spectra of phases α, β of** ***p*-Tol_2_S_2_**


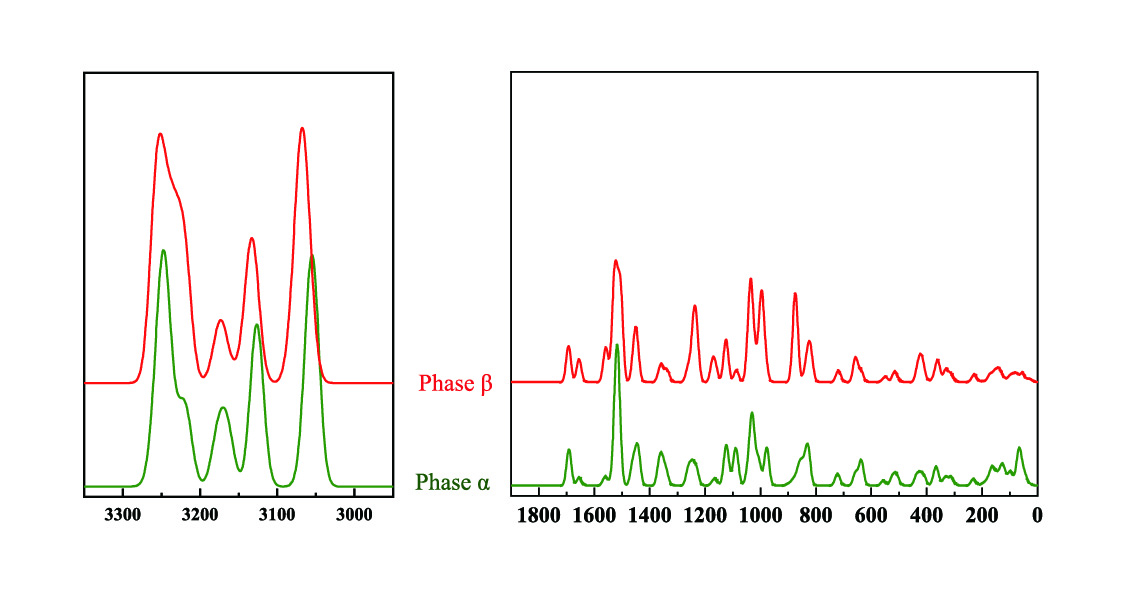


**Fig. S3** Calculated Raman spectra of phase α (green), phase β (red) of *p*-Tol_2_S_2_ under standard atmospheric pressure.

**Calculated IR Spectra of phase α, β of *p*-Tol_2_S_2_**


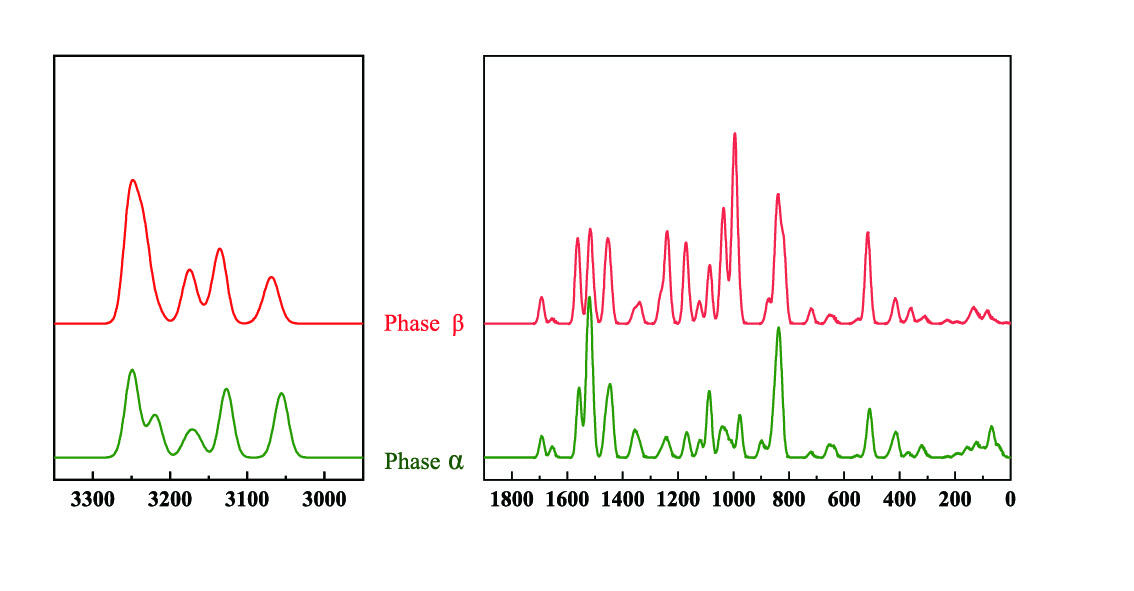


**Fig. S4** Calculated Raman spectra of phase α (green), phase β (red) of *p*-Tol_2_S_2_ under standard atmospheric pressure.

**Calculated Raman Spectra of phases α, β of *p*-Tol_2_S_2_, with different pressures**

**_
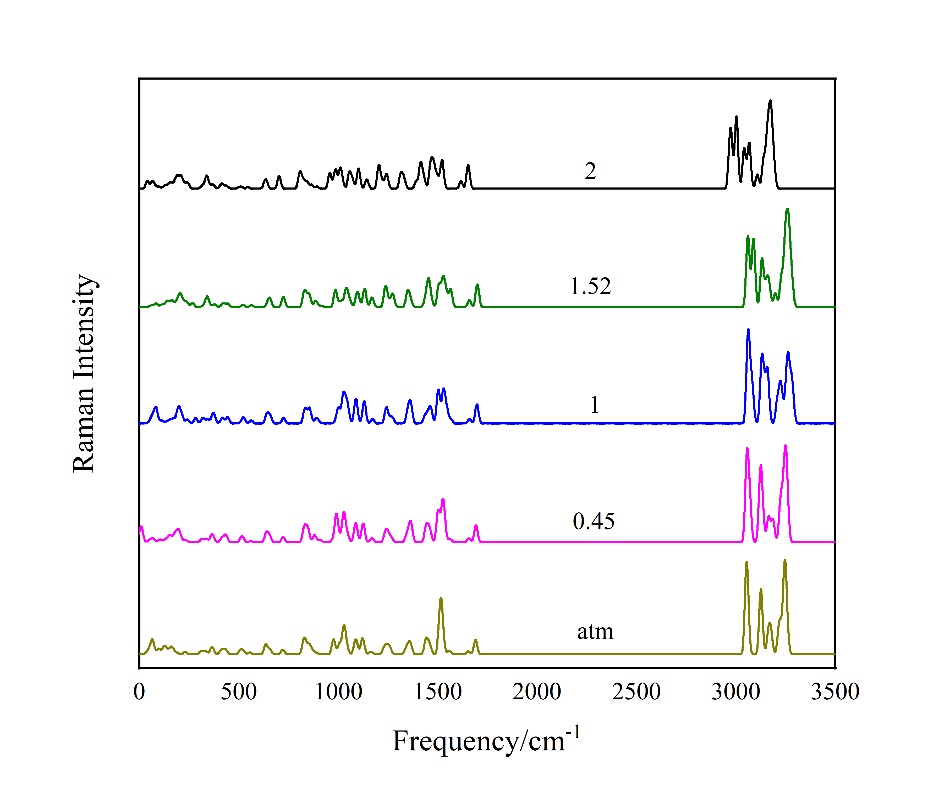
_**

**Fig. S5** Calculated Raman spectra of phase α with different pressures. The pressures from top to bottom are: 2GPa, 1.52GPa, 1GPa, 0.45GPa and atmospheric pressure, respectively.

**_
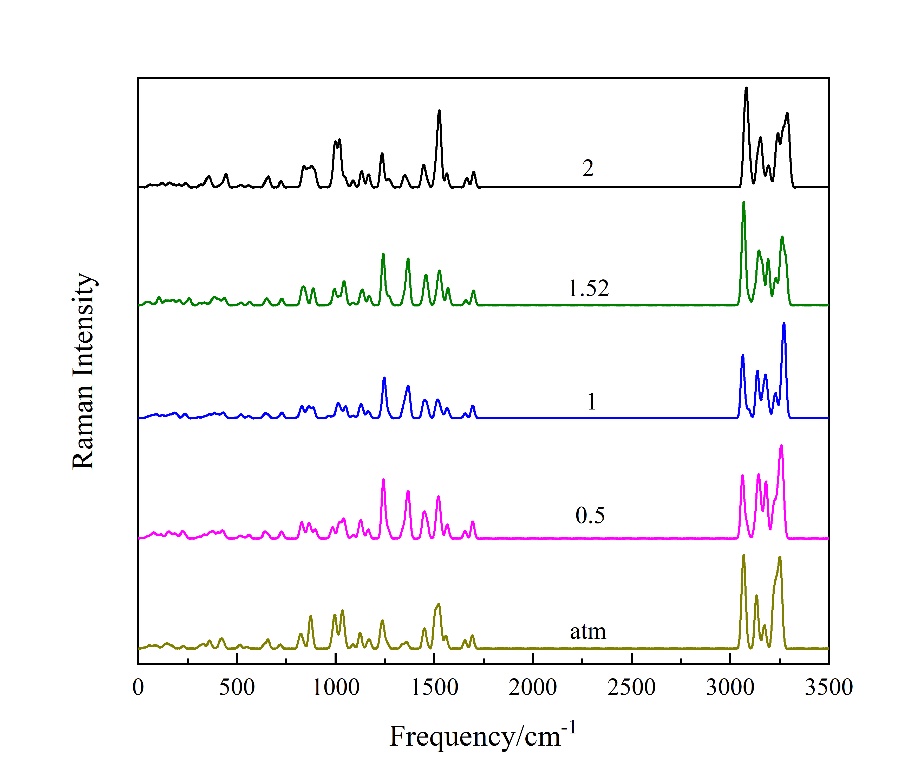
_**

**Fig. S6** Calculated different pressure Raman spectra of phase β, different color lines from top to bottom represent different pressure is 2GPa, 1.52GPa, 1GPa, 0.5GPa and atmospheric pressure, respectively.

**Calculated IR Spectra of phases α, β of *p*-Tol_2_S_2_ with different pressures**

**
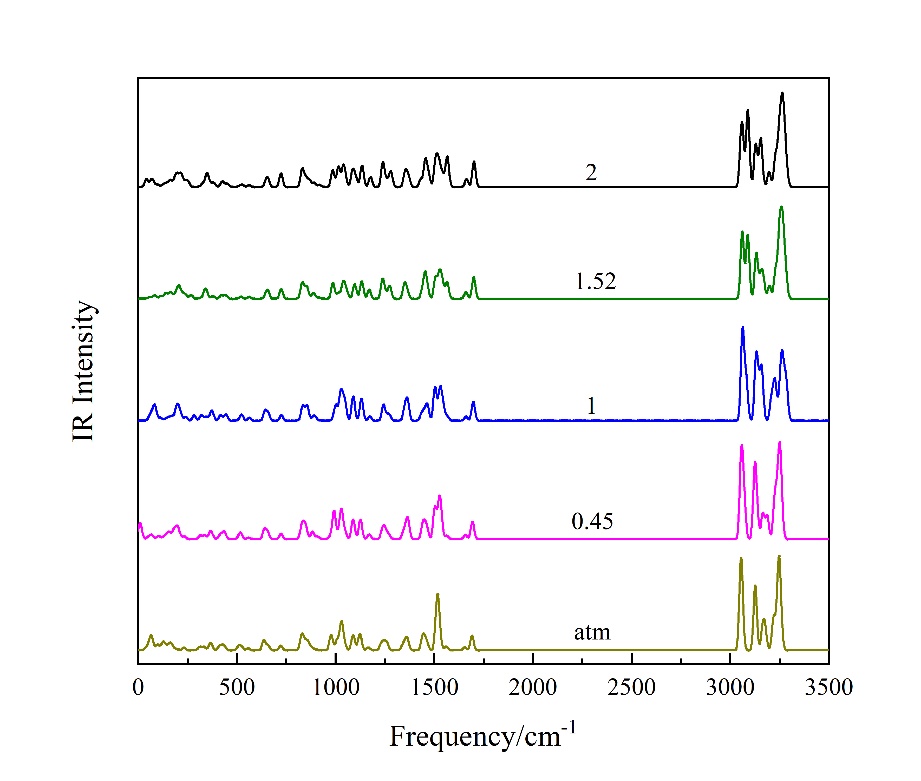
**

**Fig. S7** Calculated IR spectra of phase α with different pressures. Different color curves from top to bottom represent different pressure that are: 2GPa, 1.52GPa, 1GPa, 0.45GPa and atmospheric pressure, respectively.


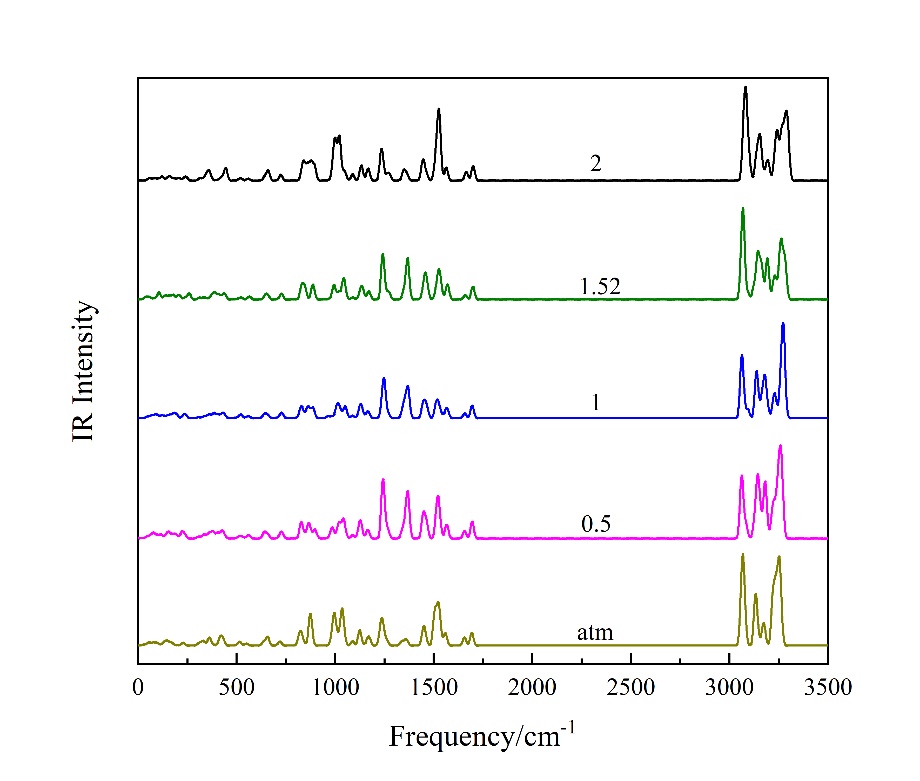


**Fig. S8** Calculated IR spectra of phase β with different pressures. Different color curves from top to bottom represent different pressure that are 2GPa, 1.52GPa, 1GPa, 0.5GPa and atmospheric pressure, respectively.

**REFERENCES:**

1. Landrum, G., RDKit: Open-source cheminformatics. Release 2014.03.1. *Zenodo*. http://dx.doi.org/10.5281/zenodo.10398
